# Serum estradiol level according to dose and formulation of oral estrogens in postmenopausal women

**DOI:** 10.1038/s41598-021-81201-y

**Published:** 2021-02-11

**Authors:** Soo-Min Kim, Sung Eun Kim, Dong-Yun Lee, DooSeok Choi

**Affiliations:** Department of Obstetrics and Gynecology, Samsung Medical Center, Sungkyunkwan University School of Medicine, 81 Irwon-ro, Gangnam-gu, Seoul, 06351 Republic of Korea

**Keywords:** Diseases, Endocrinology, Medical research

## Abstract

This study was performed to evaluate serum estradiol level in postmenopausal women using oral menopausal hormone therapy (MHT) with different doses and formulations of estrogens. A total of 344 postmenopausal women who received oral MHT was included in this cross-sectional study. Serum estradiol level was compared according to formulation (estradiol hemihydrate [EH] or valerate [EV], conjugated estrogen [CE]) and dose (estradiol 1 or 2 mg, CE 0.45 or 0.625 mg) of the estrogens. Mean age and years since menopause were 56.9 and 7.9 years, respectively. Mean duration of MHT was 27.4 months. Since serum estradiol levels were not significantly different at either dose, EH and EV at the same dose were combined for comparisons: estradiol 1 mg and 2 mg. The serum estradiol level with estradiol 2 mg (107.6 pg/mL) was significantly higher by 60% than with estradiol 1 mg (65.8 pg/mL) or CE 0.45 mg (60.1 pg/mL), and it was also significantly higher than with CE 0.625 mg (76.8 pg/mL). Our findings suggest that serum estradiol level is not directly proportional to estrogen dose. In terms of serum concentration, CE 0.45 mg is equivalent to estradiol 1 mg.

## Introduction

Menopausal hormone therapy (MHT) is the gold standard for relief of vasomotor symptoms (VMS)^1^. Although there are concerns about adverse events such as breast cancer, thrombosis, or stroke, the risk–benefit ratio of MHT is favorable for younger healthy postmenopausal women aged < 60 years or for those within 10 years since menopause onset^1–3^, and it is not recommended to routinely discontinue MHT in women aged > 60 or 65^1^.

Various formulations and doses of estrogens are used for MHT in clinical practice. MHT reduces VMS in a dose-dependent fashion. Although no significant difference was found in the effects on relieving VMS according to formulation of estrogen, such as 17β-estradiol or conjugated estrogen (CE)^1^, different profiles of estrogens might present different side effects as well as different beneficial effects on other clinical outcomes such as cognition^4^. Under these circumstances, it is important to understand relative potencies of estrogens according to formulation and dose.

Relative potencies across estrogen formulations and doses have been determined based on clinical or metabolic parameters such as relief of hot flashes, serum follicle-stimulating hormone (FSH) level, liver protein, cholesterols, or bone. However, these parameters are indirect, whereas direct measurement of serum estradiol level could be a useful method for comparing estrogen potencies.

Several small pharmacokinetic studies have evaluated serum estradiol level according to dose, formulation, or route of administration in postmenopausal women^5–11^. However, equivalent doses of different oral estrogen formulations, especially in the lower doses of estrogen that have been favored in recent years, are not clear. This study was performed to evaluate serum estradiol level in postmenopausal women using oral MHT with different formulations and doses of estrogens and to determine equivalent doses between 17β-estradiol and CE.

## Materials and methods

### Study population

All postmenopausal women who received MHT and measurement of serum estradiol level before and after MHT at Samsung Medical Center, Seoul, Republic of Korea, from 2015 to 2018 were considered for this retrospective cross-sectional study. Menopause was clinically defined as absence of spontaneous menstruation for at least one year.

Women were excluded if they (1) changed MHT regimen within three months; (2) had used a non-oral MHT regimen; (3) had used MHTs containing an estrogen component other than estradiol hemihydrate (EH), estradiol valerate (EV), or CE; (4) had a history of bilateral oophorectomy; (5) had known liver or kidney disease; (6) had a history of malignancy; (7) had a history of premature ovarian insufficiency; (8) were current smokers; (9) had excessive alcohol intake (> 10 g/day); or (10) used any other medications that might affect serum estradiol level.

Finally, 344 postmenopausal women were included for analyses. The study protocol was approved and informed consent was exempted by the Institutional Review Board of Samsung Medical Center.

### Assessments

Characteristics of the study population, including age, body mass index, menstrual history, reproductive history, past medical history, and habits of smoking and alcohol intake, were obtained from their medical records. In addition, results of laboratory tests including liver function, kidney function, and hormones were obtained.

Women were recommended to take MHT before sleep and draw the blood sample in the morning after overnight fasting. Serum estradiol level was measured using the ADVIA Centaur enhanced E2 immunoassay kits (Siemens, Tarrytown, NY, USA) following the manufacturer’s directions. Detectable concentrations ranged from 11.8 to 3000 pg/mL. The intra- and inter-assay coefficients of variation were 5.6% and 1.9%, respectively. Serum FSH level was measured using the chemiluminescent immunoassay method (Siemens, Tarrytown, NY, USA). The intra- and inter-assay coefficients of variation were 2.0% and 1.2%, respectively.

### Statistical analysis

All statistical analysis was executed using SAS version 9.4 (SAS Institute Inc., Cary, NC, USA) and R 3.5.3 (The R foundation, Vienna, Austria; http://www.R-project.org/). Data are presented as mean ± standard deviation or number (percent).

Clinical characteristics, serum estradiol, and FSH level were compared according to formulation and dose of estrogens. Categorical variables were analyzed using the Chi-square test. For continuous variables, differences between the groups were analyzed using analysis of variance followed by least significant difference post hoc test after assessing normality. A *P* value < 0.05 was considered statistically significant.

### Ethics approval

All procedures performed in studies involving human participants were in accordance with the ethical standards of the institutional and/or national research committee and with the 1964 Helsinki declaration and its later amendments or comparable ethical standards.

### Informed consent

Informed consent was exempted by the Institutional Review Board.

## Results

Table 1 presents MHT regimens included in the present study. One-hundred eight women used MHT containing EH, and the numbers of MHT users with EV and CE were 64 and 172, respectively. Various progestogens were added for endometrial protection if the woman had a uterus.

**Table 1:** Regimens of menopausal hormone therapy included in the study.

Type of estrogen	Estrogen dose (mg)	Numbers	Formulations
Estradiol hemihydrate (EH)	1	89	EH alone EH + dydrogesterone EH + drospirenone EH + norethindrone acetate
	2	19	EH alone EH + norethindrone acetate
Estradiol valerate (EV)	1	10	EV alone EV + norethindrone acetate
	2	54	EV alone EV + cyproterone acetate
Conjugated estrogen (CE)	0.45	58	CE + bazedoxifene
	0.625	114	CE alone CE + micronized progesterone

Since serum estradiol levels of EH and EV were not significantly different at the two doses (64.6 vs. 76.3 pg/mL for 1 mg and 104.5 vs. 108.8 pg/mL for 2 mg), the estrogen doses of EH and EV were combined for comparisons: estradiol 1 mg (n = 99) and 2 mg (n = 73).

In the study population, the mean age and years since menopause were 56.9 and 7.9 years, respectively. Mean duration of MHT was 27.4 months. Table 2 demonstrates the characteristics of the study population according to estrogen dose in each type of estrogen. Clinical characteristics, including age, age at menopause, body mass index, and duration of hormone therapy, did not differ for all comparisons. In addition, liver and kidney function were within the normal ranges showing no difference across all groups.

**Table 2:** Characteristics of the study population.

Variables	CE 0.45 mg (N = 58)	CE 0.625 mg (N = 114)	E2 1 mg (N = 99)	E2 2 mg (N = 73)
Age (years)	57.4 ± 5.2	56.0 ± 4.7	57.5 ± 5.8	56.9 ± 5.1
Age at menarche (years)	13.1 ± 1.1	13.4 ± 1.3	13.1 ± 1.3	13.5 ± 1.3
Age at menopause (years)	50.6 ± 3.8	49.3 ± 4.5	48.9 ± 4.7	47.6 ± 5.4
Years since menopause (years)	7.0 ± 5.0	6.8 ± 5.1	8.9 ± 5.7	9.4 ± 7.3
Duration of hormone therapy (months)	23.6 ± 18.1	25.0 ± 16.6	30.4 ± 37.4	30.0 ± 28.0
Body mass index (kg/m^2^**)**	22.2 ± 2.8	21.5 ± 2.4	21.8 ± 2.6	22.0 ± 2.0
Obese (≥ 25)	5 (8.6)	8 (7.0)	5 (5.1)	6 (8.2)
Overweight (23–24.9)	10 (17.2)	20 (17.5)	13 (13.1)	12 (16.4)
Parity (n)	1.5 ± 0.9	1.6 ± 1.0	1.7 ± 0.8	1.8 ± 1.0
AST (U/L)	22.8 ± 6.2	22.0 ± 7.6	21.7 ± 6.6	22.0 ± 9.1
ALT (U/L)	19.3 ± 7.0	18.2 ± 9.5	19.3 ± 9.7	19.6 ± 10.2
eGFR (mL/min)	87.8 ± 12.5	87.5 ± 13.8	83.8 ± 13.5	87.6 ± 16.3

Data are presented as mean ± SD or number (percent).

No significant difference was found for all comparisons.

*E2* estradiol, *AST* aspartate aminotransferase, *ALT* alanine aminotransferase, *eGFR* estimated glomerular filtration rate.

**Figure 1:**
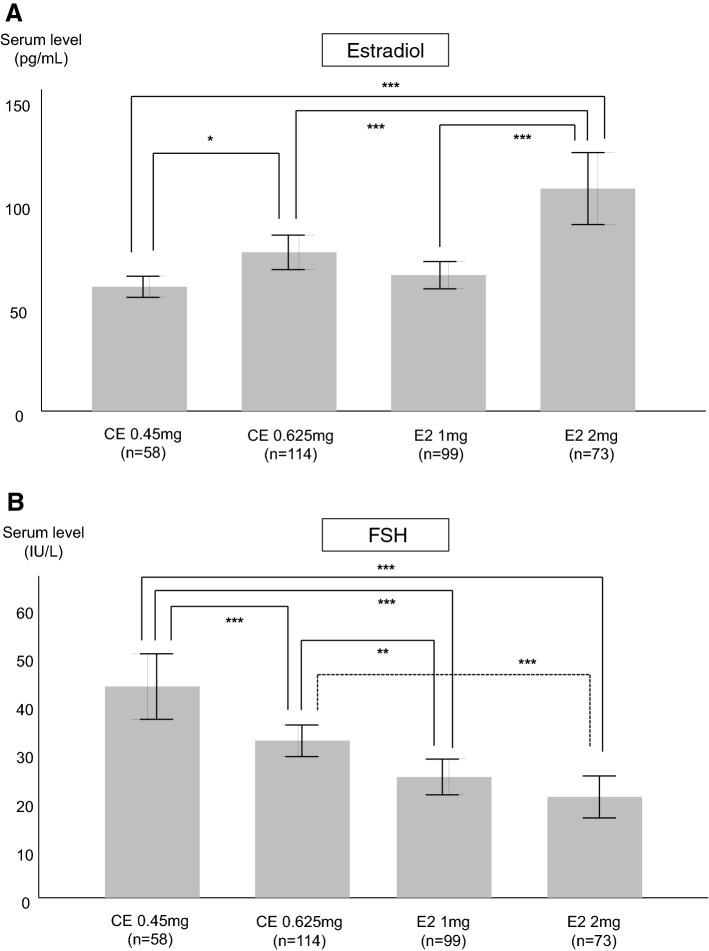
Comparisons of (**A**) serum estradiol and (**B**) serum follicle-stimulating hormone (FSH) levels according to doses and formulations of estrogens. *P < 0.05, **P < 0.01, and ***P < 0.001, using the analysis of variance followed by least significant difference post hoc test.

Figure 1 shows comparisons of serum estradiol and FSH levels according to doses and formulations of estrogens. In both estradiol and CE, serum estrogen level was significantly higher with higher estrogen doses (2 mg for estradiol and 0.625 mg for CE). The serum estradiol level with estradiol 2 mg (107.6 pg/mL) was significantly higher by 60% than with estradiol 1 mg (65.8 pg/mL) or CE 0.45 mg (60.1 pg/mL), and it was also significantly higher than with CE 0.625 mg (76.8 pg/mL) (Fig. 1A). For FSH, serum level was lower with higher estradiol and CE doses compared to lower doses. However, the mean serum level was not significantly different between the 1 and 2 mg doses of estradiol, and that of the CE 0.45 mg dose was significantly higher compared with either dose of estradiol (Fig. 1B).

## Discussion

In the present study evaluating serum estradiol level in postmenopausal women using MHT with different doses and formulations of estrogens, serum estradiol level with MHT using EH or EV was similar to previous studies reporting estrogen circulating levels of 30–50 pg/mL for estradiol 1 mg and 60–110 pg/mL for estradiol 2 mg^12,13^. However, although serum estradiol level increased with dose of estrogen, amount of increase was not directly proportional to dose of estrogen; in particular, for oral estradiol, doubling the estrogen dose from 1 to 2 mg presented an increase of approximately 60% instead of doubling the serum estradiol level. This finding suggests that ‘low-dose’ estrogen might be adequate as an initial MHT. Then, clinicians can do upward titration based on clinical response^3,14^.

For a long time, CE 0.625 mg has been the most widely used MHT and is considered the ‘standard dose’ of MHT. Generally, CE 0.625 mg is thought to be equivalent to 1–2 mg of estradiol, which is in accordance with our study showing that the serum estradiol level of CE 0.625 mg (76.8 pg/mL) remains between that of 1 mg (65.8 pg/mL) and 2 mg (107.6 pg/mL) of estradiol.

Although the optimal range for serum estradiol level to achieve therapeutic efficacy has not been established, a serum estradiol level of 60 pg/mL is needed to prevent osteoporosis^15^ and reduce 50% of hot flashes^16^. However, 0.45 mg of CE (serum estradiol level of 60.1 pg/mL in the current study) was similarly effective for treating VMS compared to 0.625 mg of CE in a randomized controlled trial^17^, which suggests that CE 0.45 mg is not inferior to CE 0.625 mg in terms of the effects on VMS. In addition, in a recent study, worsening of VMS when MHT was changed from 1 mg of estradiol to tissue-selective estrogen complex (TSEC) occurred in 16.7% of users, which was significantly lower than the rate of worsening of VMS when MHT changed from 2 mg of estradiol to TSEC (41.7%)^18^. When putting these findings together and considering that the lowest effective dose of MHT should be used to treat VMS and prevent bone loss^1^, TSEC containing 0.45 mg of CE and 20 mg of bazedoxifene could be the best initial MHT option for healthy postmenopausal women with an intact uterus.

In addition to serum estradiol level, serum FSH level also has been used to address the relative potencies of estrogens. In the present study, however, serum FSH level did not differ significantly between 1 and 2 mg doses of estradiol (20.9 vs. 25.0 IU/L), in contrast to serum estradiol level. Of interest, whereas the serum estradiol level was similar, serum FSH level was significantly higher in CE 0.45 mg (43.8 IU/L) than estradiol 1 mg (20.9 IU/L). Since CE is a mixture of estrogens, effects of CE on serum FSH level might be different compared with estradiol, even in the similar serum estradiol level. Considering that associations between serum FSH levels and surrogate markers or clinical outcomes of several diseases have been demonstrated recently, further investigation might be needed to explain the difference in responses to MHT for serum FSH level and serum estradiol level.

Although EV 1 mg is equivalent to estradiol 0.76 mg and EH and estradiol are identical, serum levels of estradiol were not significantly different between EV and EH in the present study. It might result from a small number of subjects, and a further large-scale study is warranted to draw a clear conclusion.

The present study has several strengths. First, this study was performed in a single center, and serum estradiol level was measured using the same kit. This allowed for direct comparisons of serum estradiol levels with different doses and formulations of estrogens. Second, the present study included only young healthy postmenopausal women who are the main candidates for MHT. Pharmacokinetics of oral estrogens may differ between younger and older postmenopausal women due to decrease in liver and kidney function, cardiac output, pulmonary function, muscle mass, and body composition associated with aging^19,20^. Finally, we considered many variables that could affect serum estradiol level. Age and body mass index were addressed, and women with a history of bilateral oophorectomy, premature ovarian insufficiency, known liver or kidney disease, or malignancy were excluded. Current smokers, heavy alcohol drinkers, and users of any medication that could affect serum estradiol level were also excluded from analyses.

However, our findings should be interpreted with caution. First, mass spectrometry, which can determine serum estradiol level more accurately, was not used in our study. However, the aim of this study was to compare relative potencies of various regimens based on the same method for measurement, not to determine estradiol concentration exactly. Second, differences in serum estradiol level do not directly reflect differences in the effects of MHT. Although serum estradiol concentration is closely correlated with the effects of estrogen on various tissues^21,22^, actions of each estrogen can differ according to target tissue, and a single measurement of serum estradiol level for general comparisons of ‘estrogenicity’ might be questionable. Indeed, potency of hormones is determined by interactions with receptors and response elements on the DNA and by intracellular concentration^23^, not solely by serum estradiol level. Third, effects of CE might not absolutely depend on serum estradiol level, because CE is a mixture of estrogens and major components of CE are estrone sulfate and equilin sulfate. It also might be difficult to measure serum estradiol concentrations in CE users. Fourth, pharmacokinetics of MHTs such as maximal peak or 24-h change of serum estradiol concentrations are unknown, and serum estradiol level was measured at a single time point. In addition, only drugs available in our country were included, and our results could not be extrapolated to other doses, formulations, or administration routes of estrogens. Finally, we did not consider type or dose of progestogen. However, progestogens do not affect the serum estradiol level^24^.

## Conclusion

This study suggests that mean serum estradiol level is not directly proportional to estrogen dose, and CE 0.45 mg is equivalent to estradiol 1 mg based on serum estradiol level. Our findings could be helpful when initiating or changing MHT in the clinical practice.
